# Neurocognitive outcome in children with sickle cell disease after myeloimmunoablative conditioning and haploidentical hematopoietic stem cell transplantation: a non-randomized clinical trial

**DOI:** 10.3389/fneur.2024.1263373

**Published:** 2024-05-22

**Authors:** Suzanne Braniecki, Elliott Vichinsky, Mark C. Walters, Shalini Shenoy, Qiuhu Shi, Theodore B. Moore, Julie-An Talano, Susan K. Parsons, Allyson Flower, Anne Panarella, Sandra Fabricatore, Erin Morris, Harshini Mahanti, Jordan Milner, Robert C. McKinstry, Christine N. Duncan, Carmella van de Ven, Mitchell S. Cairo

**Affiliations:** ^1^Department of Pediatrics, New York Medical College, Valhalla, NY, United States; ^2^Department of Pediatrics, UCSF Benioff Children’s Hospital, Oakland, CA, United States; ^3^Department of Pediatrics, Washington University, St Louis, MO, United States; ^4^Department of Epidemiology, New York Medical College, Valhalla, NY, United States; ^5^Department of Pediatrics, University of California, Los Angeles, Los Angeles, CA, United States; ^6^Department of Pediatrics, Medical College of Wisconsin, Milwaukee, WI, United States; ^7^Department of Medicine and Pediatrics, Tufts Medical Center, Boston, MA, United States; ^8^Department of Radiology, Washington University, St Louis, MO, United States; ^9^Dana-Faber/Children’s Cancer and Blood Disorders Center, Boston, MA, United States; ^10^Department of Medicine, New York Medical College, Valhalla, NY, United States; ^11^Department of Pathology, New York Medical College, Valhalla, NY, United States; ^12^Department of Microbiology and Immunology, New York Medical College, Valhalla, NY, United States; ^13^Department of Cell Biology and Anatomy, New York Medical College, Valhalla, NY, United States

**Keywords:** pediatric, sickle cell disease, transplant, cognitive functioning, processing speed

## Abstract

**Background:**

Due to the risk of cerebral vascular injury, children and adolescents with high-risk sickle cell disease (SCD) experience neurocognitive decline over time. Haploidentical stem cell transplantation (HISCT) from human leukocyte antigen-matched sibling donors may slow or stop progression of neurocognitive changes.

**Objectives:**

The study is to determine if HISCT can ameliorate SCD-associated neurocognitive changes and prevent neurocognitive progression, determine which specific areas of neurocognitive functioning are particularly vulnerable to SCD, and determine if there are age-related differences in neurocognitive functioning over time.

**Methods:**

We performed neurocognitive and neuroimaging in SCD recipients following HISCT. Children and adolescents with high-risk SCD who received parental HISCT utilizing CD34^+^ enrichment and mononuclear cell (T-cell) addback following myeloimmunoablative conditioning received cognitive evaluations and neuroimaging at three time points: pre-transplant, 1 and 2 years post-transplant.

**Results:**

Nineteen participants (13.1 ± 1.2 years [3.3–20.0]) received HISCT. At 2 years post-transplant, neuroimaging and cognitive function were stable. Regarding age-related differences pre-transplantation, older children (≥13 years) had already experienced significant decreases in language functioning (*p* < 0.023), verbal intelligence quotient (*p* < 0.05), non-verbal intelligence quotient (*p* < 0.006), and processing speed (*p* < 0.05), but normalized post-HISCT in all categories.

**Conclusion:**

Thus, HISCT has the potential to ameliorate SCD-associated neurocognitive changes and prevent neurocognitive progression. Further studies are required to determine if neurocognitive performance remains stable beyond 2 years post-HISCT.

**Clinical trial registration:** The study was conducted under an investigator IND (14359) (MSC) and registered at clinicaltrials.gov (NCT01461837).

## 1 Introduction

Sickle cell disease (SCD) is an autosomal recessive genetic disorder affecting an estimated 100,000 patients in the US and another 220,000 worldwide born each year (1, 2). SCD is caused by a point mutation which disrupts the structure and flexibility and promotes cellular dehydration, which leads to the significant complications of SCD, including severe pain, acute chest syndrome, chronic organ damage, cerebral vascular injury, poor quality of life, and early mortality (1–4). Improved supportive care, use of hydroxyurea therapy, and red blood cell transfusion therapy have resulted in improved childhood clinical outcomes and survival (5, 6).

Despite these improvements, cerebral vascular injury continues to be associated with significant neurological impairments in patients with SCD. The neurological impairments associated in patients with SCD have been well-documented by our group and others (7, 8). Chronic anemia and decreased oxygen supply are key features of the pathophysiology of SCD and are associated with compensatory increase in cerebral blood flow velocity and directly with cognitive issues. Specifically, chronic anemia can increase the risk of silent cerebral infarction (SCI), which can disrupt neural circuits and affect cognitive processing, potentially leading to lower cognitive skills (9).

Cognitive deficits that have been identified in children with SCD, including patients with and without a documented stroke or silent cerebral infarction, include lower overall intelligence, spatial/constructional deficits, attention and memory weaknesses, working memory issues, lower academic functioning, poor social skills, and abnormal executive functioning (7). Processing speed is a particularly vulnerable domain, with deficits mediating difficulties across other domains (10). Processing speed deficit has previously been associated with increased markers of cerebrovascular damage and represents evidence of accelerated brain damage (11). Stotesbury et al. suggested that reduced processing speed in SCD patients is correlated with hematologic differences that compromise white matter tissue integrity (12). In addition, neurological abnormalities and cognitive impairment often worsens with age in patients with SCD (8).

Neuropsychological testing with the SCD population is very important to document such cognitive issues. Pediatric neuropsychological evaluations routinely employ batteries of tests aimed at discerning strengths and deficits in broadly defined domains of functioning specific cognitive tests for use with the sickle cell population has not been established. As a result, recommendations for tests are typically extrapolated from non-SCD populations. Evidence-based practices for cognitive assessments are well-established in other populations (i.e., pediatric cancer) and are endorsed practices in professional societies, such as American Academy of Pediatrics and American Academy of Neurology. As an example, the DIVERGT is a screener utilizing well-normed, established, and widely used neuropsychological subtests which was developed to target assessment to neurocognitive domains typically affected by cranial radiation and antimetabolite chemotherapy in cancer patients (13). The Children’s Oncology Group (COG) also utilizes an abbreviated, standardized test battery that has been successfully administered in children with other chronic illnesses (i.e., specific types of leukemia and brain tumor diagnoses enrolled in the COG protocol ALTE07C1) (14). These specific domains (working memory, processing speed, executive functions, and fine motor) have also been identified as areas of deficit in patients with SCD. Evidence supports test–retest reliability, sensitivity and specificity, and discriminative and predictive validity of DIVERGT during treatment and survivors of pediatric cancer. Studies using cognitive measures in SCD have used similar measures (15).

Human leukocyte antigen (HLA)-matched sibling allogeneic hematopoietic stem cell transplantation (AlloSCT) and haploidentical stem cell transplant (HISCT) are both forms of stem cell transplantation used in sickle cell disease [i.e., AlloSCT involves obtaining stem cells from a donor who is genetically similar but not identical to the recipient (i.e., can be a sibling, unrelated volunteer, or less commonly, a cord blood unit); with HISCT, the donor is usually a half-matched family member, typically a parent, sibling, or child]. The donor is genetically only half-identical to the recipient. Stem cell transplantation is one of the only demonstrated treatments in patients with SCD that has led to improved health and neurocognitive functioning as reported by our group (16, 17). We previously reported the overall outcome, quality of life, cardio-pulmonary function, long-term donor chimerism, and immune reconstitution in children with high-risk SCD following HISCT (16, 18–20); this study is a sub-analysis of our prior Phase II clinical trial. However, its use has been limited by risk of transplantation-related morbidity and mortality and lack of HLA-matched sibling donors who also do not have homozygous SCD. Despite the limitations, patients undergoing HLA-matched sibling AlloSCT have had good neurological outcomes such as lack of new ischemic lesions, reduced cerebral velocities, and stability or improvement in cognitive functioning over time as described by our group and others (21–26). HISCT employs treatments that can reduce the risk for rejection and GVHD.

Cognitively, in a study examining cognitive functioning before AlloSCT, SCD patients had lower full scale intelligence quotient (IQ) scores and lower processing speed (23). Post-AlloSCT testing for 5 years found increased IQ and processing speed in SCD patients (23). A recent study evaluating cognition and brain magnetic resonance imaging (MRI) findings indicated stabilization of IQ and central nervous system (CNS) outcomes after unrelated donor SCT despite previous CNS abnormalities (24). Prussien et al. also found improvement in processing speed after AlloSCT (25). Similar investigations have not been done in pediatric patients undergoing HISCT.

The objective of this report was to describe changes in neuroimaging and neurocognitive sequelae in children with high-risk SCD before and following HISCT. We tested the hypothesis that host myeloimmunoablative conditioning (MIAC) followed by HISCT utilizing CD34^+^ enrichment and mononuclear cell addback in patients with high-risk SCD would result in stable neurological and neurocognitive function due to the treatment’s limiting SCD-related organ damage by donor red blood cell engraftment and chimerism.

## 2 Materials and methods

### 2.1 Patients

Eligible patients aged ≥2.0 years and ≤ 20.99 years who were homozygous for hemoglobin S with one or more high-risk factors (i.e., overt stroke, multiple acute chest syndromes, silent stroke, abnormal transcranial doppler, and multiple vaso-occlusive crises) were treated, as previously described (16). Patients received MIAC and familial HISCT utilizing CD34 enrichment and mononuclear cell addback (2×10^5^ CD3/kg) and tacrolimus single agent acute graft-versus-host disease prophylaxis, as we have previously reported [Supplementary Figure S1; (16)].

The study was approved by the institutional review board at each participating institution. Patients and/or their guardians signed written informed consents and/or assents if age applicable.

The study was conducted under an investigator IND (14359) (MSC) and registered at clinicaltrials.gov (NCT01461837).

### 2.2 Neurocognitive battery

Participants were evaluated with a combination of a well-established neurocognitive screener (DIVERGT) and abbreviated, standardized cognitive and behavioral test battery [Table 1; (13)]. The battery also consists of parent and self-report (when developmentally appropriate) measures administered at three time points: pre-transplant, 1 and 2 years post-transplant. All included measures demonstrate adequate reliability and validity for clinical use. Normative data for all included neuropsychological measures approximates the demographic distribution of the United States based on U.S. census data. Testing was conducted in yearly intervals to minimize practice effects.

**Table 1:** Neuropsychological test cognitive test battery (DIVERGT/COG-ALTE07C1).

Age ranges (years) Test	2:0–4:11	5:0–5:11	6:0–16:11	17:0–17:11	18:0–34:11 18:0
*Intelligence*					
WPPSI-IV (Vocabulary, Block Design) (15 min)	X^*^ (2:6 to 5:11)				
WISC-V (Vocabulary, Block Design) (15 min)			X		
WAIS-IV (Vocabulary, Block Design) (15 min)				X	X
*Processing speed*					
WPPSI-IV (Bug Search, Cancellation) (10 min)	X (4:0 to <6:0)				
WISC-V (Symbol Search, Coding) (10 min)			X		
WAIS-IV(Symbol Search, Coding) (10 min)				X	X
*Memory*					
CMS (Story Memory, Faces, Dot Location) (15 min)		X	X		
CVLT-C (15 min)		X	X		
WISC-V (Digit Span) (5 min)			X		
WAIS-V (Digit Span) (5 min)				X	X
WMS-IV (Logical Memory, Faces, Visual Rep) (15 min)				X	X
CVLT-III (15 min)				X	X
*Executive function*					
Trail Making Test (5 min)			X	X	X
*Language*					
Verbal Fluency (COWAT) (5 min)		X	X	X	X
Fine Motor					
Grooved Pegboard (5 min)		X	X	X	X
*Parent forms*					
COG Language Preference Questionnaire	X	X	X	X	
*Behavior/Emotion function*					
BASC-3 (20 min)	X	X	X	X	X^†^
*Executive function*					
BRIEF-P (5 min)	X	X			
BRIEF-2(5 min)			X	X	
BRIEF-A (5 min)					X^†^
*Adaptive function*					
ABAS-III (15 min)	X	X	X	X	X^†^

Subtests from the Wechsler Tests (Wechsler Primary Preschool Scales of Intelligence^,^ 4th Edition, WPPSI-IV; Wechsler Intelligence Scales for Children, 5th Edition, WISC-V; Wechsler Adult Intelligence Scales, 4th Edition, WAIS-IV; Wechsler Memory Scale, 4th Edition, WMS-IV), Children’s Memory Scale, California Verbal Learning Test (CVLT-child and adult), Trail Making Test, Controlled Oral Word Association (COWAT-verbal fluency), Grooved pegboard, Behavior Assessment Scale for Children, 3rd Edition (BASC-3), Behavior Rating Inventory of Executive Functioning, and Adaptive Behavioral Assessment Scales, 3rd Edition (ABAS-III) were used. The version of the test administered was dependent on the age of the participant. There are strong correlations between the child and adult versions, justifying their inclusion in the same analyses. Data were compared to published normative data. The test batteries were administered by a licensed psychologist, neuropsychologist, or psychometrician. Standard scores (M = 100, SD = 15), scaled scores (M = 10, SD = 3), and T scores (M = 50, SD = 10) were derived for each variable depending on the measures used to assess the domain areas and were compared across time points. The Vocabulary subtest (crystalized intelligence) from the Weschler Scales of Intelligence (WPPSI-IV, WISC-V, and WAIS-IV) was used to measure verbal IQ functioning. The Block Design subtest from the Weschler Scales of Intelligence (WPPSI-IV, WISC-V, and WAIS-IV) was used to measure non-verbal IQ functioning. The Coding and Symbol Search subtests, which comprise the Processing Speed Index from the WISC-V and WAIS-IV were used to measure processing speed. Delayed verbal memory subtests from the Children’s Memory Scale and WMS-IV were used to measure memory. Language functioning (verbal fluency) was measured with the Controlled Oral Word Association Test. Dominant hand performance on the Grooved Pegboard was used to measure fine motor skills. Behavioral functioning was measured with the BASC-3 Behavioral Symptom Index score. Attention/executive functioning was measured with the Global Executive Composite score on the Behavior Rating Inventory of Executive Functioning, which is a parent and self-report measure. ^*^For patients < 4:0: administer Receptive Vocabulary, for patients ≥ 4:0: administer vocabulary. ^†^Patients ≥18 years of age will complete a self-report form. No parent report will be used for patients 18 and older. WPPSI-IV, Wechsler Preschool and Primary Scale of Intelligence-4th Edition; WISC-V, Wechsler Intelligence Scales for Children-5th Edition; WAIS-IV, Wechsler Adult Intelligence Scales-4th Edition; CMS, Children’s Memory Scale; CVLT-C, California Verbal Learning Test—Children’s Version; CVLT-III, California Verbal Learning Test-3rd Edition; WMS-IV, Wechsler Memory Scale-4th Edition; BASC-3, Behavior Assessment System for Children-3rd Edition; BRIEF-P, Behavior Rating Inventory of Executive Function-Preschool Version. BRIEF-2, Behavior Rating Inventory of Executive Function Scales, 2nd Edition; ABAS-III, Adaptive Behavior Assessment System-3rd Edition; yrs, years.

### 2.3 Measures

The DIVERGT is comprised of six subscales from four, well-established, standardized performance-based measures of neuropsychological functioning (Trail Making Test, Controlled Oral Word Association Test [COWAT-verbal fluency], Digit Span, Symbol Search, Coding [from Weschler Tests], and Grooved Pegboard) (13). Evidence supports test–retest reliability, sensitivity and specificity, and discriminative and predictive validity of DIVERGT during treatment and survivors of pediatric cancer.

Participants’ neuropsychological functioning was further assessed using an abbreviated, standardized test battery that has been successfully administered in children with other chronic illnesses (i.e., specific types of leukemia and brain tumor diagnoses enrolled in the Children’s Oncology Group protocol ALTE07C1) (14). This test battery includes measures of intellectual functioning (Weschler Primary Preschool Scales of Intelligence, 4th Edition, WPPSI-IV; Weschler Intelligence Scales for Children, Fifth Edition, WISC-V; Weschler Adult Intelligence Scales, Fourth Edition, WAIS-IV), memory (Weschler Memory Scales, Fourth Edition, WMS-IV; Children’s Memory Scale, CMS), and also consists of parent- and self-report questionnaires that measure attention/executive functioning, adaptive functioning, and emotional/behavioral functioning (Behavioral Rating Inventory of Executive Functioning, Second Edition, BRIEF-2; Adaptive Behavior Assessment System, Third Edition, ABAS-III; Behavior Assessment System for Children, Third Edition, BASC-3). The version of the test administered was dependent on the age of the participant. There are strong correlations between the child and adult versions, justifying their inclusion in the same analyses. Data were compared to published normative data. The test batteries were administered by a licensed psychologist, neuropsychologist, or psychometrician. See Table 1 for details. Results of quality of life assessment were reported previously, showing significant improvement at 2 years of emotional and physical quality of life (20).

### 2.4 Neuroimaging

Evaluable patients had a baseline MRI and follow-up MRIs at 1 and 2 years post-HISCT. Robert C. McKinstry, MD, PhD, neuroradiologist, conducted a central blinded review of the imaging scans and provided case report forms for each patient, per MRI imaging guidelines previously discussed (Supplementary Methods S1). Measurement of global atrophy, infarctions, CNS hemorrhage, and cerebral vascular injury were previously defined by DeBaun et al. (27).

### 2.5 Statistical methods

Descriptive statistics were computed for each domain score at each time point (baseline, 1 year post, and 2 years post). Two-way analysis of variance, repeated measures analysis (mixed effects) was used to assess differences between time points using the GraphPad Prism statistical program (Prism, GraphPad Inc., San Diego, CA, United States). A difference score was used to analyze data across multiple time points as an index of change over time or the difference between two measures using the same sampling unit. Analyses were conducted between age groups (12.9 and under, 13.0 and over) to assess for any age-related change in scores from baseline to the 1- and 2-year assessment. *p* values less than 0.05 are considered significant and represent an improvement. Stabilization of scores was defined as little statistical variation in the scores over time. This sub-analysis was planned before the beginning of the study, which represented one of the secondary objectives.

## 3 Results

There were 21 patients who met eligibility for HISCT and were consented on the study. Two patients had peripheral blood stem cells collected and CD34^+^ enrichment performed from their parental haploidentical donor but later withdrew consent prior to the start of conditioning secondary to social issues. Both are still alive being treated with supportive care. The remaining 19 patients proceeded to HISCT. The age (mean ± standard error of mean) (range) of the 19 HISCT recipients was 13.1 ± 1.2 years (3.3–20.0). The gender ratio was 12/7 male/female. One patient refused to participate in the neurocognitive portion of the study. The resultant evaluable patients were as follows: 10 patients aged 12.9 and younger and eight patients 13 years and older at the baseline assessment. Of the 18 patients tested at baseline, 14 patients were tested at year 1 and 12 patients were tested at year 2 (related to patient deaths, lost to cognitive follow-up). Participant flow and testing diagram is noted in Supplementary Figure S2. Table 2 summarizes study baseline characteristics. The overall event-free survival following HISCT was 90% at 1-year post-transplant, as we have previously reported [Supplementary Figure S3; (16)]. Furthermore, hemoglobin recovery at year 1 and year 2 were improved over time and were found to be in the normal range, and hemoglobin S versus A reflected donor S trait (Supplementary Figures S4A,B, respectively). Finally, whole blood and red cell donor chimerism at 1 year was 97.1 ± 1.4% and 96.4 ± 2.0%, respectively (Supplementary Figures S5A,B).

**Table 2:** Clinical characteristics and clinical outcome results of HISCT recipients (*N* = 19).

Pt #	Age yr	M/F	HLA Match Out of 6	Primary risk factor	Neurological status at HISCT	Neutrophil engraftment Day +	Platelet engraftment Day +	Status Day +	Baseline MRI changes Yes/No	1 yr MRI imaging changes Yes/No	2 yr MRI imaging changes Yes/No	Baseline cognitive deficits Yes/No
526–001	10	M	3/6	TCD	WNL	13	33	A/3805	No	No	No	Yes
526–002	13	F	3/6	Stroke	WNL	9	16	A/3692	Yes	No	No	Yes
526–003	20	F	4/6	Stroke	WNL	9	19	A/3398	Yes	No	No	Yes
526–004	18	F	3/6	ACS	WNL	10	NE	D/59	No	Deceased	--	Yes
526–005	20	M	3/6	VOC	WNL	9	12	A/3188	No	Yes	No	Yes
526–006	12	M	3/6	VOC	WNL	9	44	A/3168	Yes	No	No	Yes
526–007	8	M	3/6	Silent infarct	WNL	11	21	A/3119	Yes	No	No	Yes
526–008	9	F	3/6	Stroke	WNL	9	17	A/3014	Yes	Yes	No	Yes
526–009	15	M	3/6	Stroke	WNL	6	8	A/2884	Yes	Yes	No	Yes
526–010	4	M	3/6	ACS	WNL	10	15	A/2520	No	No	No	Yes
526–011	17	F	3/6	Silent infarct	WNL	9	NE	D/141	No	Deceased	--	Yes
526–012	14	M	3/6	TCD	WNL	9	90	A/2786	No	No	No	Yes
526–013	12	F	3/6	Stroke	WNL	9	14	D/390	Yes	No	Deceased	Yes
526–014	20	F	3/6	Stroke	WNL	9	18	A/2713	Yes	No	No	Yes
526–015	10	M	3/6	ACS	WNL	10	33	A/2531	No	No	No	No
526–016	20	M	3/6	Stroke	WNL	10	19	A/2349	No	No	No	Yes
526–018	3	M	3/6	ACS	WNL	10	16	A/2352	No	No	No	No
526–019	11	M	3/6	Silent infarct	WNL	10	33	A/2083	Yes	No	No	No
526–020	11	M	3/6	Silent infarct	WNL	9	8	A/2076	Yes	No	No	No
Summary mean ± SEM	13 ± 1.2	12/7 M/F	3/6 *N* = 18 4/6 *N* = 1	N/A	N/A	9.5 ± 0.3	24.5 ± 4.7	Med f/u 2,884 days (59–3,805)	N/A	N/A	N/A	N/A

HISCT, haploidentical stem cell transplantation; N, number; Pt, patient; yr, year; M/F, male/female; HLA, human leukocyte antigen; MRI, magnetic resonance imaging; TCD, transcranial Doppler abnormal; A, alive; ACS, acute chest syndrome; NE, not engrafted; D, death (004, VOC, 011, AGVHD, 013, CGVHD); VOC, venoocclusive disease; N/A, not applicable; SEM, standard error of the mean; f/u, follow-up; pre-existing cognitive deficits defined as weakness in one or more areas on testing (intellectual, processing speed, memory, language, and motor), using standardized guidelines, (SS < 70, ss < 7).

### 3.1 Neuroimaging results

At baseline, nine out of evaluable 19 patients had evidence of an infarct (one patient did not have a baseline MRI), one patient with CNS hemorrhage, four patients with cerebral atrophy, and six patients with evidence of cerebral vascular injury (five patients had more than one type of event). At 1-year post-transplant, one individual out of 16 patients had a new infarct, one individual patient with new CNS hemorrhage and one individual patient with progressive atrophy, and no patients had new cerebral vascular injury. At 2 years post-transplant, there were no new overt and/or silent infarcts, no new CNS hemorrhage, no new global atrophy, and no new cerebral vascular injury (Table 3; patient details in Supplementary Table S1).

**Table 3:** Neuroradiology results (number of events for evaluable patients).

	Infarcts	CNS hemorrhage	Cerebral atrophy	Cerebral vascular injury
Baseline	9/18	1/18	4/18	6/18
1 Year^*^	1/16	1/16	1/16	0/16
2 Year^*^	0/14	0/14	0/14	0/14

^*^New findings compared to baseline. CNS, central nervous system.

### 3.2 Cognitive functioning

Eighteen evaluable patients were included in the analysis (one patient refused this portion of the study; 18 patients at baseline, 14 patients at year 1, and 12 patients at year 2). Most patients had both pre-existing cognitive deficits and neuroradiological findings (10/18 patients) at baseline testing, with one or more weaknesses in the areas of intellectual (verbal IQ and non-verbal IQ), processing speed, memory, language, and motor functioning, with weaker motor and processing speed skills being the predominant areas of weakness.

**Figure 1:**
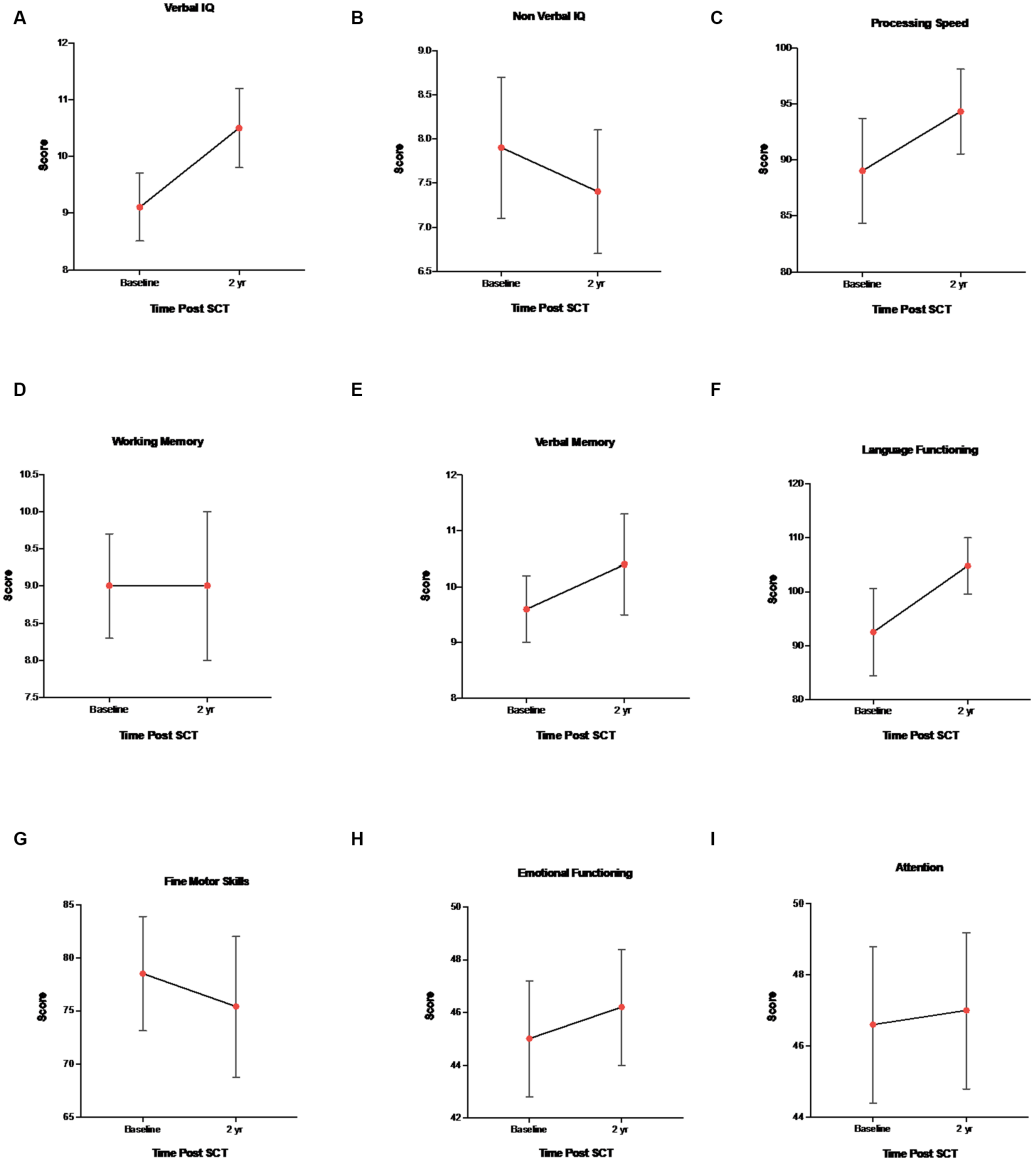
Mean ± SEM changes in neurocognitive measures. **(A)** Verbal IQ (WAIS-IV, WISC-V, WPPSI-IV, and vocabulary), **(B)** Non-verbal IQ (WAIS-IV, WISC-V, WPPSI-IV, and block design), **(C)** WAIS-IV, WISC-V, WPPSI-IV, and processing speed, **(D)** WAIS-IV, WISC-V, and working memory, **(E)** CMS, WMS-IV, verbal memory delay, **(F)** COWAT and language functioning, **(G)** Grooved pegboard and fine motor skills, **(H)** BASC-3 and emotional functioning, and **(I)** BRIEF-2 and attention/executive functioning.

Overall intellectual functioning (verbal IQ and non-verbal IQ), verbal memory, working memory, processing speed, language, and attention/executive functioning scores were within the average range at all three time points, and there were no statistically significant differences between age, gender, and time point. Behavioral and attention functioning was within the average range for baseline, 1 and 2 years post-assessment. Fine motor skills for both baseline and 2 years post-transplant were well below average, but stable over time (stability was defined as little statistical variation over time). Effect size estimates (Cohen’s d) indicated a small sized effect for non-verbal IQ, fine motor skills, verbal memory and emotional functioning, moderate sized effect for processing speed and attention, large size effect for language, and no effect size for working memory (baseline vs. year 2) (Table 4). See Figure 1 for mean ± SEM changes over time and individual changes in Supplementary Figure S6 for subject test scores at baseline versus year 2.

**Table 4:** Neurocognitive functioning changes from baseline, 1, and 2 years post-HISCT.

Time point	Verbal IQ (i.e., vocabulary)	Nonverbal IQ (i.e., block design)	Processing speed	Working memory	Verbal memory delay	Language functioning	Fine motor skills	Emotional functioning	Attention/executive functioning
Baseline	9.1 ± 0.6	8.5 ± 3.5	89.0 ± 4.7	9.0 ± 0.7	9.6 ± 0.6	92.5 ± 8.1	78.5 ± 5.4	45.0 ± 2.2	46.6 ± 2.2
1-year post-HISCT	9.6 ± 0.7	8.8 ± 3.2	88.1 ± 3.7	9.9 ± 0.6	9.8 ± 0.8	105.8 ± 4.5	75.8 ± 5.9	45.36 ± 2.9	45.1 ± 2.9
2 years post-HISCT	10.5 ± 0.7	7.4 ± 2.3	94.3 ± 3.8	9.0 ± 1.0	10.4 ± 0.9	104.8 ± 5.2	75.4 ± 6.6	46.20 ± 2.2	47.0 ± 2.2
*p* value at 1-year versus baseline	0.91	0.7	0.74	0.23	0.36	0.11	0.36	0.77	0.36
*p* value at 2 year versus baseline	0.08 (*d* = −0.433; moderate)	0.5 (*d* = 0.185; small)	0.08 (*d* = −0.592; moderate)	0.86 (*d* = 0; none)	0.27 (*d* = −0.340; small)	0.16 (*d* = −1.183; large)	0.79 (*d* = 0.288; small)	0.96 (*d* = 0.240; small)	0.14 (*d* = 0.633; moderate)

Verbal IQ Functioning (WAIS-IV, WISC-V, WPPSI-IV, and vocabulary subtest), non-verbal IQ functioning (WAIS-IV, WISC-V, WPPSI-IV, and block design subtest), working memory (WAIS-IV, WISC-V, WPPSI-IV, and digit span), verbal memory delay presented as scaled score (CMS and WMS-IV), processing speed (WAIS-IV, WISC-V, and WPPSI-IV), language (COWAT), and motor grooved pegboard presented as subtest/standard score, emotional functioning (BASC-3) presented as a T score, attention/executive functioning (BRIEF-2) presented as T score; p-values were determined by paired Student t-test using the Prism statistical program (GraphPad, San Diego, CA, United States); *p* < 0.05 is considered statistically significant (see also Figure 1). SEM, standard error of mean; *d*, Cohen’s *d* value (effect size); HISCT, haploidentical stem cell transplantation.

**Figure 2:**
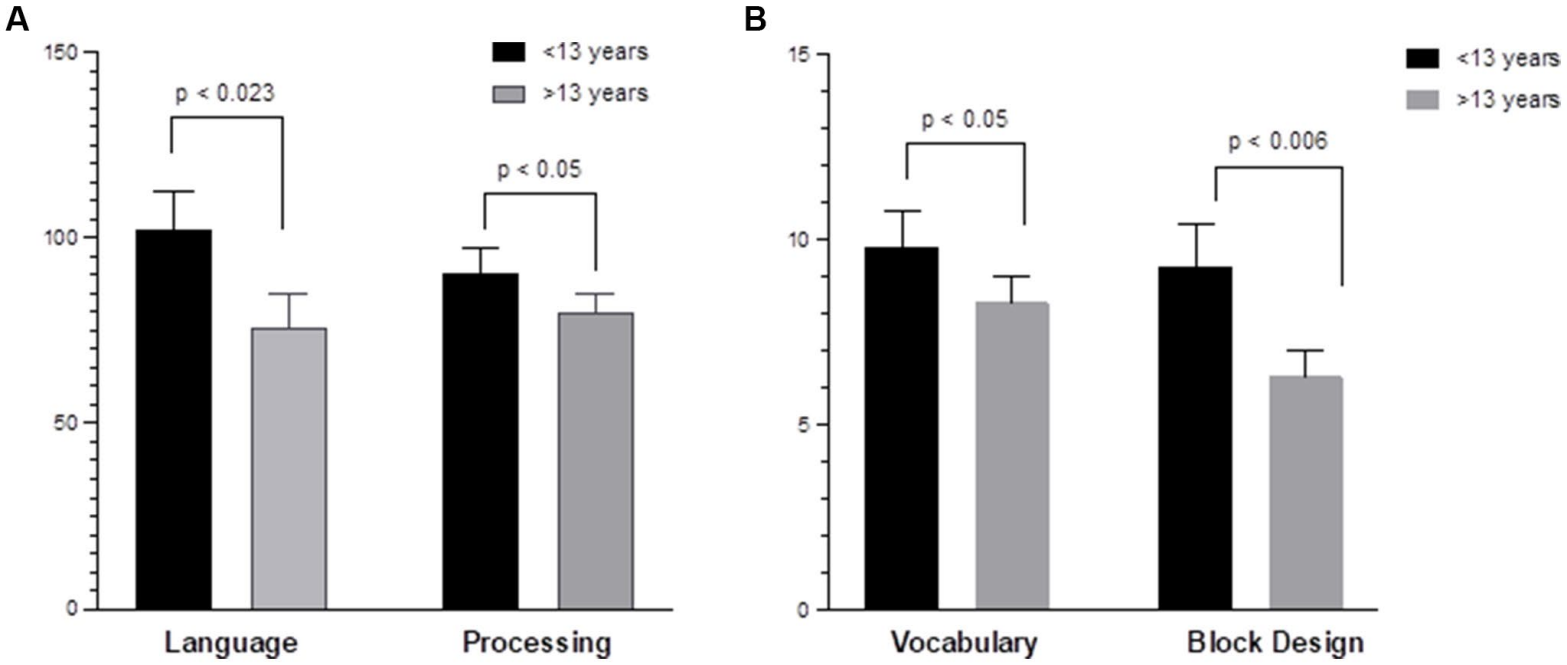
Age-related differences in neurocognitive functioning. **(A)** Younger children (<13 years) demonstrated higher baseline scores in language (COWAT) (*p* < 0.023); processing speed (WISC-V and WPPSI-IV) (*p* < 0.05) compared to older children (≥13 years) (Standard score). **(B)** Younger children demonstrated higher verbal IQ (WISC-V, WPPSI-IV, and vocabulary) (*p* < 0.05); non-verbal IQ (WPPSI-IV and WISC-V) (*p* < 0.006) compared to older children (≥13 years). (Scaled score).

**Figure 3:**
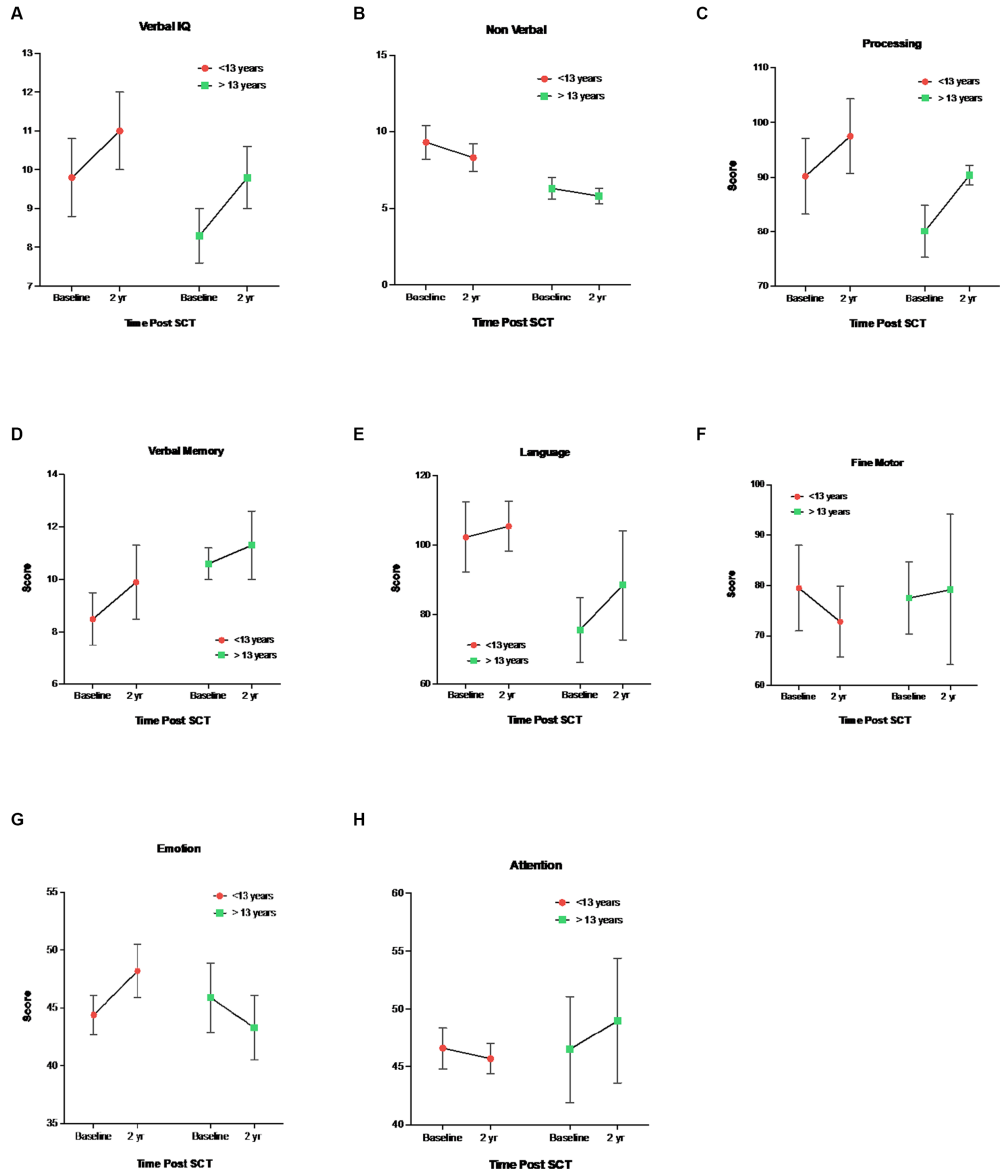
Mean ± SEM trajectories in neurocognitive functioning stratified by <13 vs ≥13 years. 1 **(A)** Verbal IQ (WAIS-IV, WISC-V, WPPSI-IV, Vocabulary), **(B)** Nonverbal IQ (WAIS-IV, 2 WISC-V, WPPSI-IV, Block Design), **(C)** Processing Speed, WAIS-IV, WISC-V, WPPSI-IV, 3 **(D)** Verbal Memory Delay, CMS, WMS-IV, **(E)** Language Functioning, COWAT, **(F)** Fine 4 Motor Skills, Grooved Pegboard, **(G)** Emotional Functioning, BASC-3, **(H)** Emotional 5 Functioning, BRIEF-2.

Regarding age-related differences, younger children (<13 years) demonstrated higher baseline (pre-HISCT) scores in language functioning (standard score) (*p* < 0.023) (Figure 2A), verbal IQ (scaled score) (*p* < 0.05) (Figure 2B), non-verbal IQ (scaled score) (*p* < 0.006) (Figure 2B), and processing speed (standard score) (*p* < 0.05) (Figure 2A) compared to older children (≥13 years). Younger children demonstrated average functioning in these areas, whereas older children performed in the borderline to low average range at baseline testing. At 1 year and 2 years post-HISCT, younger children demonstrated stability in scores, and stability was also seen for older children in these four areas. There were no statistically significant age-related differences with regard to verbal memory, fine motor skills, and emotion and attention functioning. Aggregate trajectories baseline versus 2 years <13 versus ≥13 years are highlighted in Figure 3.

Two-way analysis of variance, repeated measures test was also conducted to examine differences between patients with history of stroke (*n* = 10) and those without history of stroke (*n* = 8). There were no statistically significant findings with any of the cognitive variables.

## 4 Discussion

Results of our study indicated stable neuroimaging and neurocognitive function from baseline to 2 years post-HISCT. Hemoglobin A levels were also found to recover over time, with no patients having any SCD-related signs or symptoms after HISCT and long-term donor whole blood and RBC chimerism. Historically, both overt and silent infarcts have been associated with a significant change in full scale, verbal and performance intelligence quotients (28, 29). Our results found overall average intellectual functioning, which was stable over time. Other studies have found similar results, although the cause behind these findings remains inconclusive (30, 31).

In addition, our findings showed improved neurocognitive processing speed 2 years after HISCT. Impaired processing speed in SCD patients is one of the most major deficits in neurocognitive function and appears independent of whether there was an overt and/or silent infarct demonstrated on MRI. Reduced processing speed is likely related to chronic anemia, oxygen desaturation, and integrity of normal white matter (12). The mechanism for white matter injury is not fully understood, but cerebral hemodynamics are often abnormal, increasing the risk of ischemia.

Processing speed can also be susceptible to brain aging and can decrease in age in both normal adults and other populations in general. Adults with symptomatic SCD demonstrate worse processing speed and experience more executive functioning issues than their healthy siblings, despite treatment with hydroxyurea (12). Thus, mild improvement of processing speed at 2 years post-HISCT is quite encouraging, although further follow-up is warranted. Our group also found improved pulmonary functioning 2 years post-HISCT, which may be related to improvements in oxygen saturation and anemia (19).

We explored whether there were age-related differences in cognitive functioning to further assess impact of disease progression. In our study, age-related differences at baseline pre-HISCT were found for verbal and non-verbal IQ, language functioning, and processing speed. Younger children remained relatively stable over time from baseline to 2 years post-transplant. On the other hand, older children had significantly lower baseline scores but stabilized over time post-HISCT and demonstrated no more significant changes. Older children and adults with SCD have been reported to have lower scores than younger children across various cognitive measures, which may be the result of cumulative effects of the cerebral vascular injury within the brain.

Schatz et al. (30) found that older patients with prior infarction were more likely to have coexisting cardiovascular risk factors such as hypertension and diabetes, which contribute to the increased risk of hemorrhage.

Deficits in language functioning are also fairly common among children with SCD and appear to be specific to the neurologic effects of the disease (32–35). For example, language weaknesses in syntactical skills have been associated with cerebrovascular infarcts and high cerebral blood flow velocities, even when controlling for disease severity (34). Progression of the disease appears to play a role in age-related differences in language functioning, and school absences may also contribute to the development of language functioning in SCD patients (35). The receptive and expressive language skills of 10 children with strokes due to SCD were significantly poorer than those of their matched controls (33).

In regard to our findings, older children may have experienced greater neurologic events than younger children which may have led to weaker baseline performance. Over time, without progression of neurological events, they were able to recover which may also be related to improved processing speed, allowing for more efficient language retrieval. In addition, older children may have experienced more school absences due to SCD-related symptoms which may have compromised their language development. Return to school may have positively impacted language functioning. Such results speak to the great need for early intervention (36).

Neuroimaging results indicated stable MRI scans over time, which is commensurate with other findings in studies with transplantation. King et al. reported stabilization of IQ and CNS post-HISCT despite prior CNS morbidity and neurological complications after transplant (37). In addition, Walters et al., from our group, previously reported on late effects following HLA-matched sibling donor AlloSCT with myeloablative conditioning in children with severe SCD (17). After AlloSCT, patients with stroke who had stable engraftment of donor cells experienced no subsequent stroke events after AlloSCT and brain MRI scans demonstrated stable or improved results (17).

In a study of neurologic outcomes following myeloablative conditioning and AlloSCT in patients with SCD, 41% of patients transplanted with a matched sibling donor graft for SCD had a history of cerebrovascular abnormalities on imaging before AlloSCT. The majority of patients had improvement or stabilization of their neurologic disease after AlloSCT (38). Patients who underwent neurocognitive testing also showed a statistically non-significant trend toward improvement in IQ and stabilization of IQ and nervous system outcomes after unrelated donor SCT (37).

In a study of patients who underwent matched sibling donor or haploidentical AlloSCT, no patient with sustained engraftment exhibited any clinical evidence of a stroke or progression on imaging studies by 5 years after AlloSCT. MRI, magnetic resonance angiography, and transcranial doppler studies showed improvement in white matter changes and stable or even improved vessel abnormalities. Cognitive functioning was stable after AlloSCT. Thus, overall, AlloSCT seems to limit the progression of organ toxicity from the natural history of severe SCD without AlloSCT (39).

Limitations of the study include small sample size and missing data (i.e., resulting from patient death and lost to cognitive follow-up). In addition, there was a lack of a comparison group to be able to determine if results were related to HISCT or not. Another limitation includes the use of different intelligence tests over time. While these tests are designed to assess cognitive abilities across different age groups, it is important to consider potential differences in test content, format, and administration procedures between editions. Nonetheless, these intelligence tests are widely recognized and accepted tools for longitudinal research to track changes and examine change/stability of cognitive abilities over time. Further studies are necessary to determine if neurocognitive performance remains stable longer than 2 years after the transplant and to compare the data to individuals who have not received transplant and with healthy siblings/peers, which is ongoing in this study. In addition, it will be important to compare neurocognitive outcomes in SCD patients who received HISCT versus gene therapy (40). These results should be compared to other results of HISCT studies in SCD recipients using alternative approaches.

Overall, the results of this study suggest that HISCT utilizing MIAC in high-risk patients with SCD is associated with excellent outcomes and appear to be comparable to HLA-matched sibling AlloSCT. This study also corroborates what other recent studies have found regarding stable cognition and neuroimaging post AlloSCT as we and others have previously reported (41). In addition, this study demonstrates the importance of early treatment delivery, which can decrease or stop further cognitive changes and even improve functioning. Recent guidelines from the American Society of Hematology also placed high importance on and recommended treatments aimed at preventing neurovascular disease and maintaining cognitive function (42). These results will be of significant benefit to general pediatricians and hematologists managing SCD patients.

## Data availability statement

The raw data supporting the conclusions of this article will be made available by the authors, without undue reservation.

## Ethics statement

The studies involving humans were approved by the Institutional Review Board (IRB) at New York Medical College. The studies were conducted in accordance with the local legislation and institutional requirements. Written informed consent for participation in this study was provided by the participants’ legal guardians/next of kin. Written informed consent was obtained from the individual(s), and minor(s)’ legal guardian/next of kin, for the publication of any potentially identifiable images or data included in this article.

## Author contributions

SB: Conceptualization, Data curation, Formal analysis, Investigation, Methodology, Validation, Writing – original draft, Writing – review & editing. EV: Data curation, Formal analysis, Writing – review & editing. MW: Data curation, Formal analysis, Writing – review & editing, Conceptualization. SS: Conceptualization, Data curation, Formal analysis, Writing – review & editing. QS: Data curation, Writing – review & editing, Formal analysis. TM: Data curation, Formal analysis, Writing – review & editing, Conceptualization. J-AT: Conceptualization, Data curation, Formal analysis, Writing – review & editing. SP: Conceptualization, Data curation, Formal analysis, Writing – review & editing, Writing – original draft. AF: Data curation, Formal analysis, Writing – review & editing. AP: Data curation, Formal analysis, Writing – review & editing. SF: Data curation, Formal analysis, Writing – review & editing. EM: Data curation, Formal analysis, Writing – review & editing. HM: Data curation, Formal analysis, Writing – review & editing. JM: Data curation, Formal analysis, Writing – review & editing. RM: Writing – review & editing, Writing – original draft. CD: Writing – review & editing, Data curation, Formal analysis. CV: Data curation, Formal analysis, Writing – review & editing, Writing – original draft. MC: Data curation, Formal analysis, Writing – original draft, Writing – review & editing, Conceptualization, Funding acquisition, Investigation, Methodology, Project administration, Resources, Supervision, Validation, Visualization.
