# Supplementary material for: Neurocognitive outcome in children with sickle cell disease after myeloimmunoablative conditioning and haploidentical hematopoietic stem cell transplantation: a non-randomized clinical trial
Source: Front Neurol. 2024 May 22;15:1263373. doi: 10.3389/fneur.2024.1263373 (PMC11151850; doi:10.3389/fneur.2024.1263373)
Supplement: Supplementary file 1 [file Data_Sheet_1.pdf]

Supplementary Material

Neurocognitive outcome in children with sickle cell disease after myeloimmunoablative conditioning and haploidentical hematopoietic stem cell transplantation

Suzanne Braniecki[1], Elliott Vichinsky[7], Mark C. Walters[7], Shalini Shenoy[8], Qiuhu Shi[6], Theodore B. Moore[9], Julie-An Talano[10], Susan K. Parsons[11], Allyson Flower[1], Anne Panarella[1], Sandra Fabricatore[1], Erin Morris[1], Harshini Mahanti[1], Jordan Milner[1], Robert C. McKinstry[9, 12], Christine N. Duncan[13], Carmella van de Ven[1], and Mitchell S. Cairo[1-5]

Departments of [1]Pediatrics, [2]Medicine, [3]Pathology, [4]Microbiology & Immunology, [5]Cell Biology & Anatomy, and [6]Department of Epidemiology, New York Medical College, Valhalla, NY, USA; [7]Department of Pediatrics, UCSF Benioff Children's Hospital, Oakland, CA, USA; [8]Department of Pediatrics and [12]Radiology, Washington University, St Louis, MO, USA; [9]Department of Pediatrics, University of California Los Angeles, Los Angeles, CA, USA; [10]Department of Pediatrics, Medical College of Wisconsin, Milwaukee, WI, USA; [11]Departments of Medicine and Pediatrics, Tufts Medical Center, Boston, MA, USA; [13]Dana-Faber/Children's Cancer and Blood Disorders Center, Boston, MA, USA.

Correspondence: Mitchell S. Cairo, MD
Email: mitchell_cairo@nymc.edu

1. **Supplementary Methods 1.**
   Neuroimaging Guidelines
2. **Supplementary Figures 1 to 6.**
3. **Supplementary Tables 1.**

Supplementary Methods 1. Neuroimaging Guidelines

Cranial magnetic resonance imaging (MRI) and magnetic resonance angiography (MRA) will be performed prior to Day -59 commencing conditioning for transplant according to this protocol. A similar scan will be repeated at 1 and 2 years post-transplant to evaluate change in previously existing lesions or identify and define the presence of any new onset lesions.

Study Description: MRI/MRA
Protocol Scan Time = 15 to 20 minutes
Standard Head Coil (circularly polarized or quadrature)

1. **Scout Localizer (3 planes) Time: 9 sec**

2. **Fast/Turbo FLAIR T2-weighted Axial and Coronal Acquisition** (Must cover the whole brain) Time: 5:42 min approx (for TR 9000ms)

Alignment AC-PC line (undersurface of the genu & splenium of the corpus callosum)	Echo Train Flip Angle: 180 degrees 1 acquisition
TR = 10,000ms (acceptable value 9000- 10000ms)	Matrix 256 (AP) x 192 (L-R)
TE = 125ms (acceptable range: 90ms- 130ms)	FOV 210mm (acceptable range is 210 – 230 mm)
T1 = Dependent on TR. Set to null CSF signal (acceptable range 2000-3000)<br>[for TR of 9 seconds, T1 = 2500ms; for TR of 10 seconds, T1 = 2308ms)	Slice Thickness = 5mm<br>No intersection gap is desirable (acceptable intersection gap 0 – 1mm)
Echo Train Length = 7 (acceptable range is 5 – 11)	

3. **T1-weighted Acquisition (for segmentation and volumetrics)**

<table>
<tr><td rowspan="2">Option 1:
3-D T1-weighted fast gradient echo: (MPRAGE or IRprep-3DSPGR)
Time: 7:07 min approx (for TR 1900)

Orientation = Sagittal TR= 1900 ms
TE= ~4ms

T1= 1100 ms
Slab Thickness=160mm (acceptable range = 160- 180mm)</td><td colspan="2">Option 2:
T1-weighted Sagittal and Axial Acquisitions Tiem: 1:38 min approx (for TR 500ms) for Sagittal Time: 1:38 min approx (for TR 500ms) for Axial</td></tr>
<tr><td>Orientation 1 = Sagittal (Must cover the whole brain)

TR 500ms (acceptable range is 400-800ms)</td><td>Orientation 2 = Axial (Must cover the whole brain)

Aligned to match the orientation of the FLAIR acquisition</td></tr>
<tr><td>Slab Partitions = 128 (acceptable range = 120- 144)
Slab Partition Thickness= 1.25 mm (acceptable range 1.25 – 1.5mm)</td><td>TE 12ms (use minimum value, acceptable range 10- 30ms)</td><td>TR 500ms (acceptable range is 500-800ms)</td></tr>
<tr><td>FOV= 256mm
Matrix= 222 x 256</td><td>1 acquisition
Matrix 256 (AP) x 192 (L-R)</td><td>TE 12ms (use minimum value, acceptable range 10-30ms)</td></tr>
</table>

Resolution = 1.0 x 1.0 x 1.25mm (acceptable Limit	FOV 12mm (acceptable range is<br><br>Slice Thickness = 5mm No intersection gap is desirable (acceptable	1 acquisition<br><br>Matrix 256 (AP) x 192 (L-R) FOV 210mm (acceptable range is 210-230mm)<br><br>Slice Thickness = 5mm No intersection gap is desirable (acceptable intersection gap 0- 1mm

4. **Fast/Turbo Spin Echo T2-weighted Axial Acquisition (Must cover the whole brain)**

Time: 1:16 min approx (for TR 4000ms)

Aligned to match the orientation of the FLAIR<br>TR = 5000ms (acceptable value 3000- 6000ms)<br>TE = 100ms (acceptable range: 90ms- 110ms)<br>Flip Angle = 180 degrees<br>1 acquisition	Echo Train Length = 7 (acceptable range is 5 – 11)<br>Matrix 256 (AP) x 192 (L-R)<br>FOV 210mm (acceptable range is 210 – 230 mm)<br>Slice Thickness = 5mm<br>No intersection gap is desirable (acceptable intersection gap 0 – 1mm)

Extended MRI Protocol

Extended MRI Protocol Scan time = 15 to 20 minutes (As time allows for appropriate subjects)

5. **Echo-Planar Axial Diffusion Acquisition (Repeated 2 times. Do not use signal averaging)**

Time: 45 sec (for TR 6000ms) x 2 = 1:30 min approx

Aligned to match the orientation of the FLAIR acquisition (if possible)<br>TR = 6000ms (acceptable value 3000 - 6000ms)<br>TE = 90ms (minimum value desirable, acceptable range: 75ms- 140ms)<br>1 acquisition<br>Matrix: 128x128	FOV 230mm<br>Slice Thickness = 5mm<br>No gap between slices: (must not allow an intersection gap)<br>b values: 0, 1000 s/mm^2<br>Gradient Orientation: Minimum X, Y, Z for computation of the trace of the tensor<br>Desirable: 6 or more directions to compute the tensor

6. **3D Time-of-Flight Magnetic Resonance Angiography**

Time: 9:12 min approx (for 192x512 matrix)

Axial slab centered on the supraclinoid internal carotid artery<br>MOTSA = multiple thin slab acquisitions strategy is desirable)<br>Slab partitions >60, with resolution <1mm Number of Slabs= 3<br>TE minimum (<5ms)	Flip Angle = 25 degrees, optimized for TR (TONE or variable RF pulse flip angel desirable).<br>Matrix: 192 x 512 (acceptable range up to 512 x 512)<br>FOV: minimum allowable for TE <5ms, and for head size MT pulse is acceptable to improve background suppression.<br>Venous (Cephalad) presaturation pulse

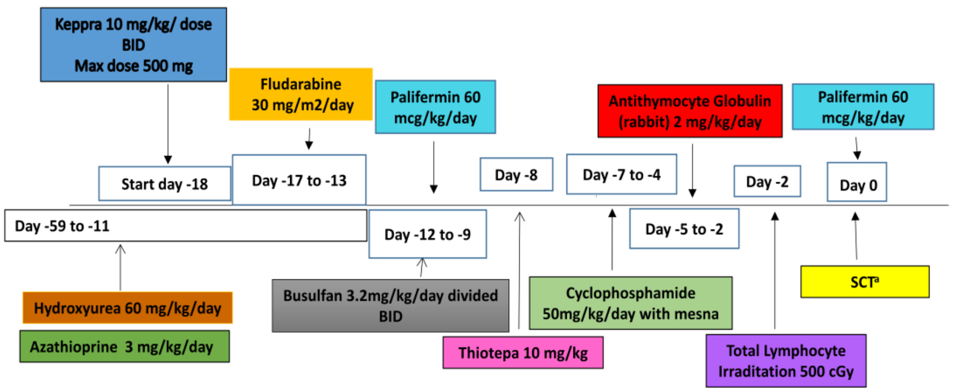

Supplementary Figure 1. Myeloimmunoablative condition regimen. Patients received hydroxyurea and azathioprine starting day -59 to day -11, fludarabine on Days - 17, -16, -15, -14, -13; busulfan twice daily on Days -12, -11, -10, -9; thiotepa on day -8; cyclophosphamide on Days -7, -6, -5, -4; TLI on day -2 rabbit ATG on day -5, -4, -3, and -2. Supportive care included RBC exchange transfusion to keep the hemoglobin S ≤30% during the 59 days of conditioning. Platelet transfusions were administered to maintain a platelet count ≥50K/mm^3 until platelet recovery. Levetiracetam was administered Day -18 through Day +100; Palifermin was administered 3 days prior to busulfan and Days 0 - +2 post-HISCT; and SOS prophylaxis on Day -17 to day +30. Routine HISCT supportive care also included granulocyte-colony stimulating factor post-HISCT and prophylaxis for herpes simplex virus, pneumocystis jiroveci pneumonia, cytomegalovirus, and fungus as we have previously described. HISCT, haploidentical stem cell transplantation.
From JAMA Pediatrics. 2020. 174(2): 195-197. Copyright© 2020 American Medical Association.
All rights reserved.

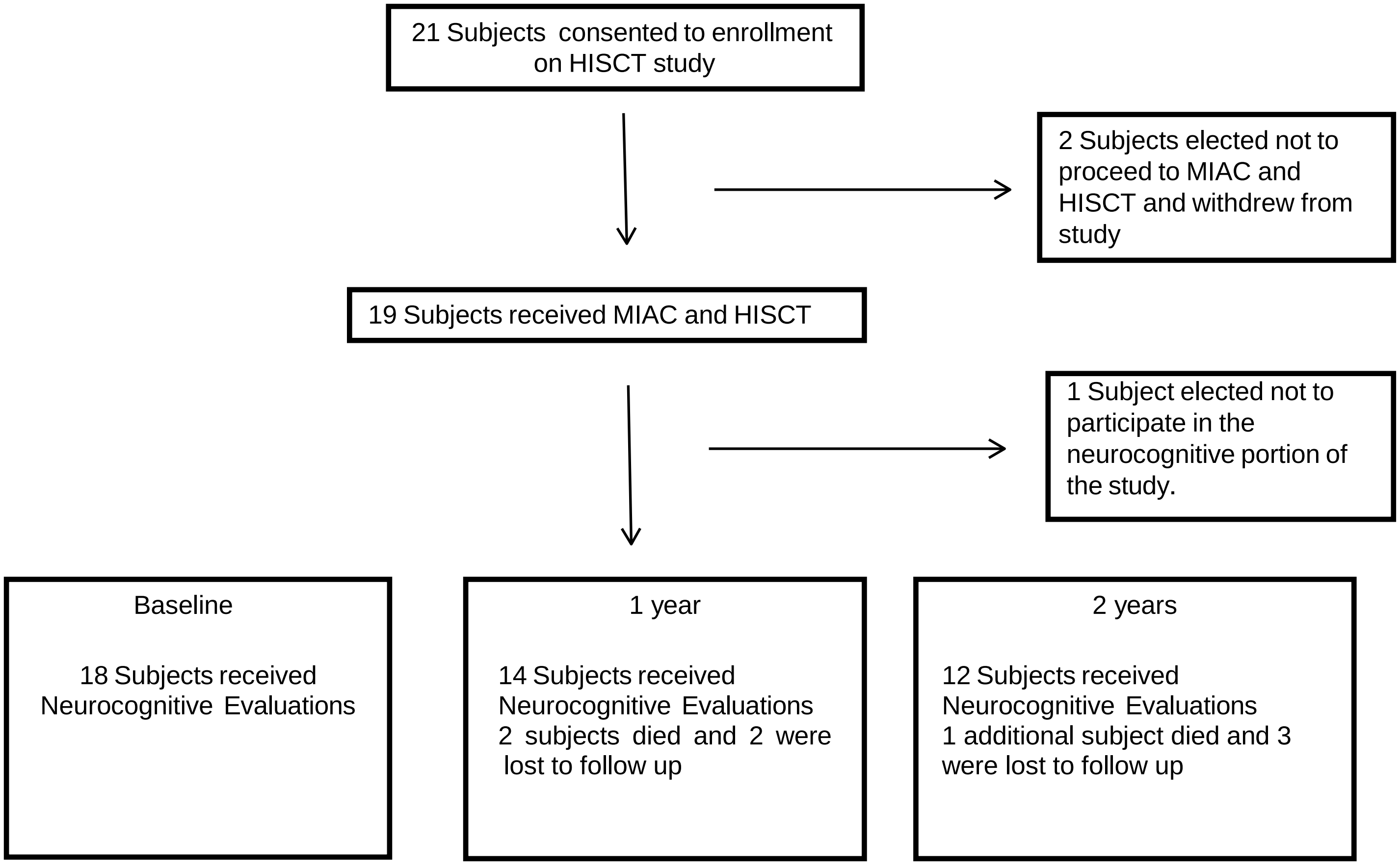

Supplementary Figure 2 Participant flow and testing diagram.

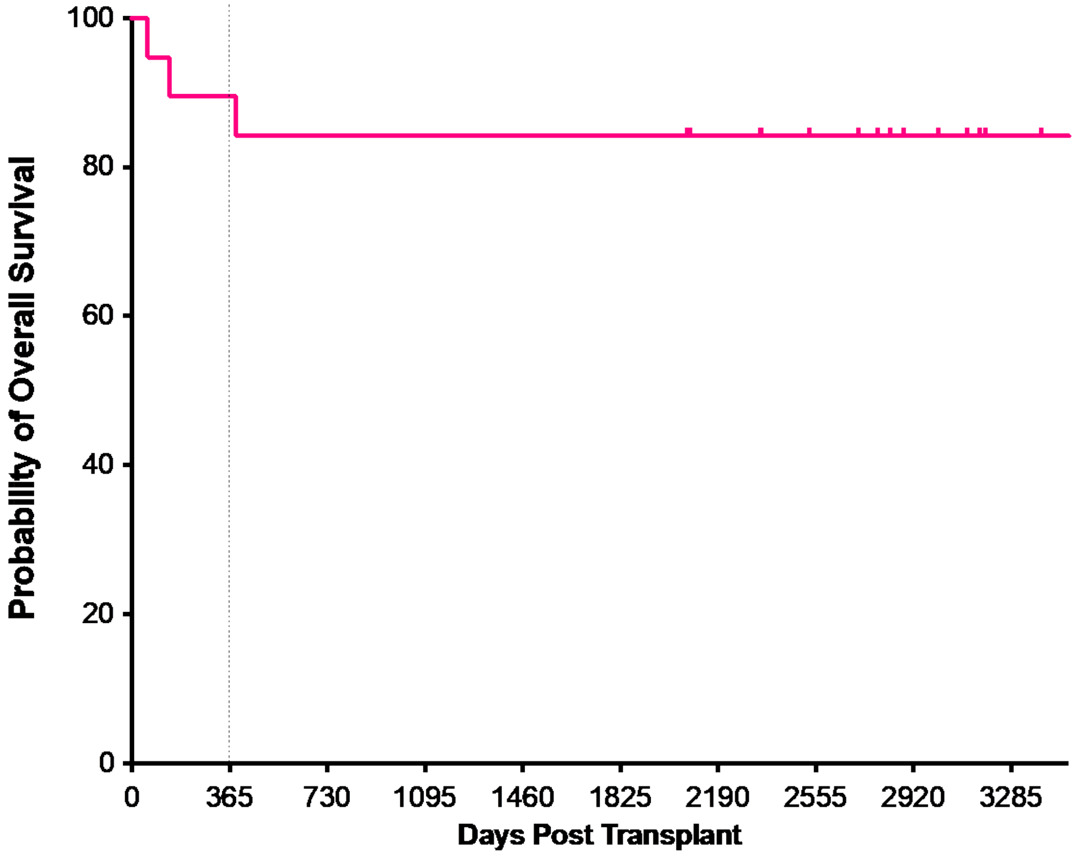

Day	0	59	141	390	1712	1719	1985	1988	2167	2349
Number at risk	19	19	18	17	16	15	14	13	12	11

Day	2422	2468	2520	2650	2755	2804	2824	3034	3328	3441
Number at risk	10	9	8	7	6	5	4	3	2	1

Supplementary Figure 3. Overall survival rate. The probability of EFS/OS in 19 enrolled patients following familial haploidentical stem cell transplantation utilizing donor $CD34^+$ enrichment and MNC addback determined by the product limit method of Kaplan and Meier. Median follow-up 2884 days (59-3805).
EFS, event free survival; OS, overall survival; MNC, mononuclear cells; HISCT, haploidentical stem cell transplantation; No, number.

A

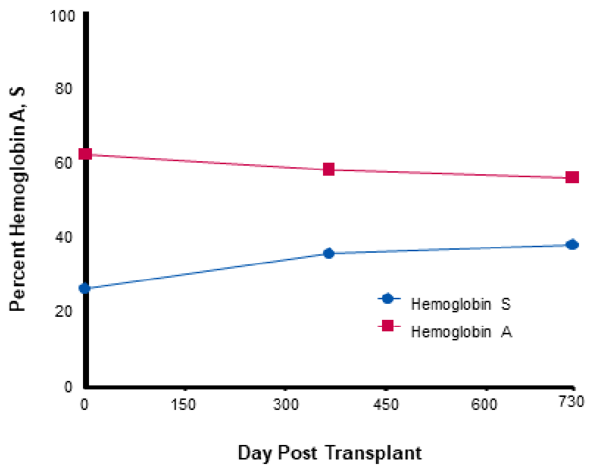

B

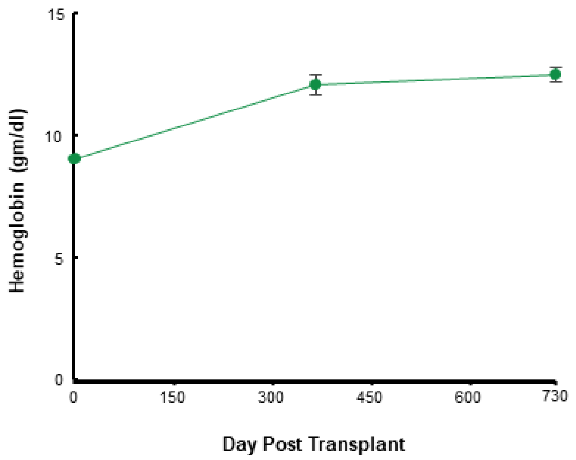

Supplementary Figure 4. Reconstitution of hemoglobin and stratified by hemoglobin A vs hemoglobin S. **(A)** The distribution of hemoglobin A vs hemoglobin S by CBC and quantitative hemoglobin electrophoresis (mean ± SEM). **(B)** Hemoglobin concentration in the SCD post-HISCT recipients at day 0, 365 and 730. CBC, complete blood count; SCD, sickle cell disease.

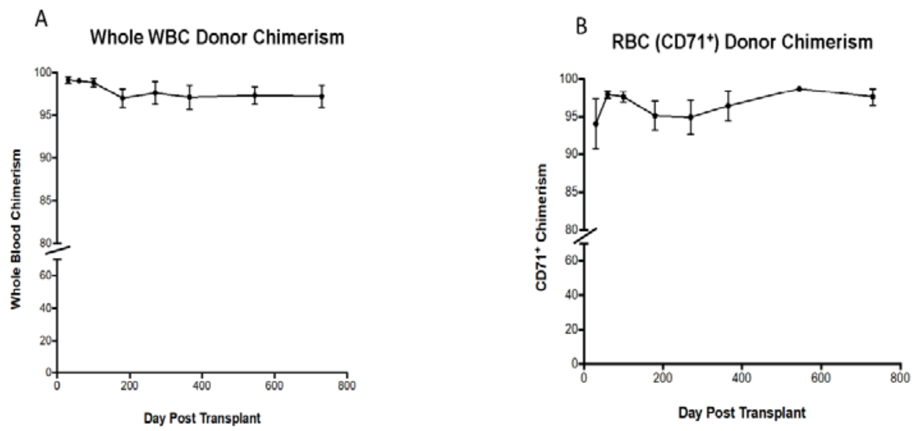

Supplementary Figure 5. Whole blood and RBC donor chimerism.

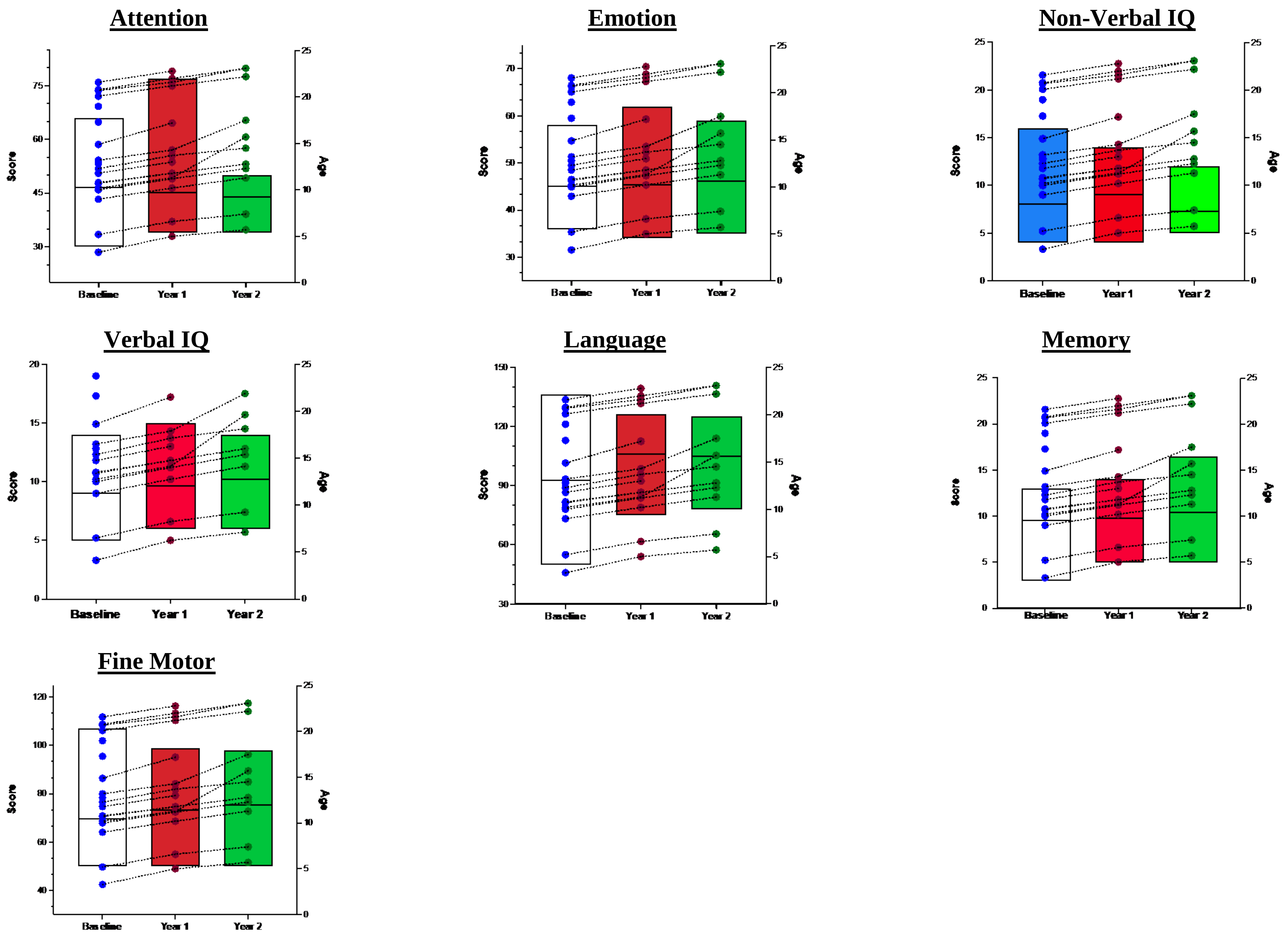

Supplementary Figure 6. Individual evaluable subject test scores (left Y axis) at Baseline (blue box), Year 1 post therapy (red box) and

Year 2 post therapy (green box); right Y axis and lines and dots indicate patient ages at same Baseline, Year 1 and Year 2 time points.

Supplementary Table 1.

MRI Findings at Baseline, 1yr and 2 yrs

Patient #	Baseline	Year 1	Year 2
001	No remarkable findings on MRI	No remarkable findings on MRI	No remarkable findings on MRI
002	5 infarct like lesions, indeterminate for cerebral vasculopathy, no hemorrhage, global atrophy present	1 infarct lesion, but stable and no other new infarct lesions, evidence of cerebral vasculopathy, global atrophy is unchanged, no hemorrhage	1 infarct lesion, but stable and no new lesions, evidence of cerebral vasculopathy, but stable from year 1, global atrophy is unchanged, no hemorrhage
003	At least 5 extensive areas of infarction, evidence of cerebral vasculopathy, no hemorrhage, no global atrophy	No new infarcts, evidence of cerebral vasculopathy, but stable, no hemorrhage	No new infarcts, evidence of cerebral vasculopathy, but stable, no hemorrhage
004	No remarkable findings on MRI	Deceased	Deceased
005	No remarkable findings on MRI	Evidence of intracranial hemorrhage, no other new findings	Evidence of intracranial hemorrhage, but stable and no other new findings
006	At least 5 infarct lesions, no evidence of intracranial hemorrhage, presence of cerebral vasculopathy, global atrophy is present	Infarct lesions, but no new findings, no evidence of intracranial hemorrhage, presence of cerebral vasculopathy, but stable, global atrophy is present, but stable	Infarct lesions, but stable and no new lesions, no evidence of intracranial hemorrhage, presence of cerebral vasculopathy, but stable, global atrophy is present, but stable
007	At least 1 infarct lesion, evidence of intracranial hemorrhage, no cerebral vasculopathy, no global atrophy	At least 1 infarct lesion- but no new lesions, evidence of intracranial hemorrhage- but stable, no cerebral vasculopathy, no global atrophy	At least 1 infarct lesion- but stable and no new lesions, evidence of intracranial hemorrhage- but stable, no cerebral vasculopathy, no global atrophy
008	At least 1 infarct lesion, no evidence of intracranial hemorrhage, evidence of cerebral vasculopathy, global atrophy is present	At least 1 infarct lesion- no new lesions, no evidence of intracranial hemorrhage, evidence of cerebral vasculopathy-but stable, global atrophy is progressive	At least 1 infarct lesion- but stable and no new lesions, no evidence of intracranial hemorrhage, evidence of cerebral vasculopathy-but stable, global atrophy is unchanged from year 1

009	No infarct lesion, no evidence of intracranial hemorrhage, evidence of cerebral vasculopathy, global atrophy is present	One infarct lesion, no evidence of intracranial hemorrhage, no evidence of cerebral vasculopathy-improved from baseline, global atrophy is unchanged	One infarct lesion-but stable from year 1 and no new, no evidence of intracranial hemorrhage, no evidence of cerebral vasculopathy, global atrophy is unchanged
010	No remarkable findings on MRI	No remarkable findings on MRI	No remarkable findings on MRI
011	Poor MRI- insufficient for determination of lesions or hemorrhage. cerebral vasculopathy, global atrophy is indeterminate	Deceased	Deceased
012	No remarkable findings on MRI	No remarkable findings on MRI	No remarkable findings on MRI
013	At least 3 infarct lesions, no evidence of hemorrhage or cerebral vasculopathy, global atrophy is present	At least 3 infarct lesions- no new lesions, no evidence of hemorrhage or cerebral vasculopathy, global atrophy is present, decreased size of bilateral subdural collections, which may represent chronic subdural hematomas, (no mention of subdural hematomas in baseline report)	Deceased
014	At least 2 infarct lesions, evidence of cerebral vasculopathy, no global atrophy, indeterminate hemorrhage	At least 2 infarct lesions- but stable and no new, improved cerebral vasculopathy, no global atrophy, no evidence of hemorrhage	At least 2 infarct lesions- but stable and no new, stable cerebral vasculopathy from year 1, no global atrophy, no evidence of hemorrhage
015	No remarkable findings on MRI	Interval development of 2 foci of T-signal loss within left cerebellum that were not seen on baseline and may represent chronic intracranial blood products, chiari malformation, unchanged, and 3 mm aneurysm arising from the region of the left superior cerebellar artery, unchanged compared to baseline MRI (no mention of these findings in baseline report)	Stable aneurysm, no new MRI findings

016	Right sided frontal temporal parietal craniectomy and craniotomy changes; encephalomalacic change seen in the right front temporal lobe with cavity change and retractive change of the adjacent right temporal horn<br><br>No other remarkable findings on MRI	No remarkable findings on MRI	No data
018	Prominent perivascular spaces about the bilateral posterior horns of the lateral ventricles with focal coalescent cystic appearing areas<br><br>No other remarkable findings on MRI	No remarkable findings on MRI	No remarkable findings on MRI
019	No remarkable findings on MRI. Evidence of cerebral vasculopathy	No evidence of infarcts, hemorrhage, atrophy. Improvement of cerebral vasculopathy	No evidence of infarcts, hemorrhage, atrophy. Improvement of cerebral vasculopathy still seen
020	Evidence of infarct, no other evidence	Stable infarct, no new findings	Stable infarct, no new findings
